# Highly Pathogenic Avian Influenza Virus (H5N1) Outbreak in Captive Wild Birds and Cats, Cambodia

**DOI:** 10.3201/eid1503.081410

**Published:** 2009-03

**Authors:** Stéphanie Desvaux, Nick Marx, Sivuth Ong, Nicolas Gaidet, Matt Hunt, Jean-Claude Manuguerra, San Sorn, Malik Peiris, Sylvie Van der Werf, Jean-Marc Reynes

**Affiliations:** Centre de Cooperation Internationale en Recherche Agronomique pour le Développement, Montpellier, France (S. Desvaux, N. Gaidet); WildAid, Phnom Penh, Cambodia (N. Marx, M. Hunt); Institut Pasteur du Cambodge, Phnom Penh (S. Ong, J.-M. Reynes); Institut Pasteur, Paris, France (J.-C. Manuguerra, S. Van der Werf); National Animal Health and Production Investigation Center, Phnom Penh (S. Sorn); University of Hong Kong and Queen Marie Hospital, Pokfulam, Hong Kong Special Administrative Region, People’s Republic of China (M. Peiris)

**Keywords:** Influenza A virus, animal, wild animals, zoo, birds, transmission, cats, Cambodia, disease outbreaks, dispatch

## Abstract

From December 2003 through January 2004, the Phnom Tamao Wildlife Rescue Centre, Cambodia, was affected by the highly pathogenic influenza virus (H5N1). Birds from 26 species died. Influenza virus subtype H5N1 was detected in 6 of 7 species tested. Cats from 5 of 7 species were probably infected; none died.

On January 24, 2004, the first confirmed outbreak of highly pathogenic avian influenza virus (HPAIV) subtype H5N1 in Cambodia was reported to the Office International des Epizooties (*1*). During the previous month, an unusually high mortality rate had been noted among captive wild birds at the Phnom Tamao Wildlife Rescue Centre (PTWRC) in Takeo Province, 45 km South from Phnom Penh. We report the results of a retrospective investigation of this outbreak.

## The Study

During the outbreak period, PTWRC housed 600–1,000 wild animals (70 species of mammals, birds, and reptiles). The center is divided into 3 main sections that cover 37 ha. Birds were kept in sections S1–1, S1–2, and S2, and the cats were in all sections (Figure). The information on bird deaths at PTWRC was systematically recorded by WildAid staff members who were at the Centre at the time of the outbreak. In June 2004, a complete investigation was conducted at PTWRC, and semistructured interviews of key informants were used to identify deaths of domestic poultry in the surrounding villages. Every bird death between December 15, 2003, through January 15, 2004, was defined as a suspected case of HPAIV (H5N1). For S1, the cumulative mortality rate could not be estimated because the exact bird population was not known and the birds were difficult to observe in that section (the semicaptive waterfowl population is able to mix with the wild population and disperse to breed). For S2, information was complete (Table 1).

**Figure:**
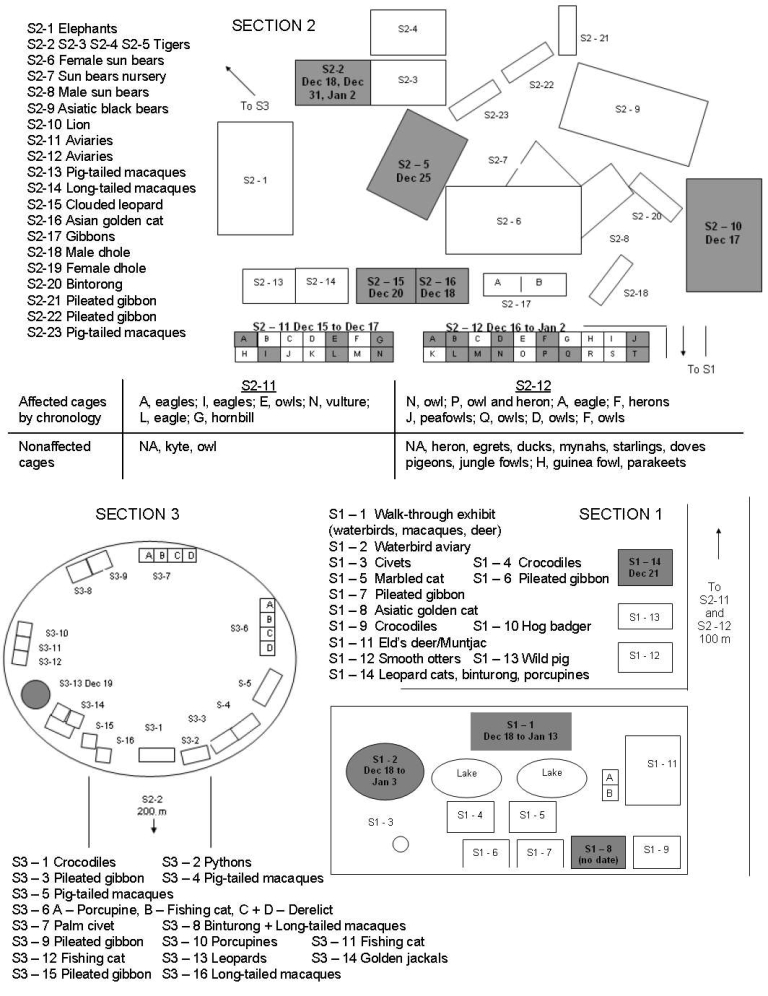
Map of the 3 main sections of the Phnom Tamao Wildlife Rescue Centre, Cambodia, during outbreak of highly pathogenic avian influenza virus (H5N1), December 15, 2003–January 13, 2004. Shaded areas indicate contaminated cages (labeled with date of outbreak). NA, exact cage not available.

**Table 1:** Cumulative deaths during an outbreak of highly pathogenic avian influenza virus (H5N1), Phnom Tamao Wildlife Rescue Centre, Cambodia, December 15, 2003–January 13, 2004*

Order	Family	Species (common name), no. sampled	No. dead birds in S1	No. birds not dead in S1	Cumulative deaths in S2, % (dead/total at risk)	
					Per species	Per order
Anseriformes	Anatidae	*Anas poecilorhyncha* (Indian spot-billed duck)	NP	NP	0 (0/4)	0 (0/4)
Ciconiiformes	Ardeidae	*Ardea cinerea* (grey heron), n = 2	4	2	NP	47 (9/19)
		*Ardeola speciosa* (Javan-pond heron), n = 1	7	0	100 (7/7)	
		*Butorides striatus* (little heron)	NP	NP	0 (0/1)	
		*Egretta garzetta* (little egret)	NP	NP	18 (2/11)	
	Ciconidae	*Ephippiorhynchus asiaticus* (black-neck stork)	1	3	NP	
		*Leptoptilos dubius* (greater adjutant stork)	2	1	NP	
		*Leptoptilos javanicus* (lesser adjutant stork)	3	21	NP	
		*Mycteria leucocephala* (painted stork)	6	20	NP	
		*Ciconia episcopus* (wooly necked stork)	0	3	NP	
		*Anastomus oscitans* (Asian openbill stork)	0	5	NP	
Colombiformes	Colombidae	*Treron curvirostra* (thick-billed green pigeon)	NP	NP	0 (0/7	0 (0/17)
		*Streptopelia chinensis* (spotted dove)	NP	NP	0 (0/10)	
Coraciiformes	Buceritidae	*Buceros bicornis* (great hornbill)	NP	NP	100 (1/1)	100 (1/1)
Falconiformes	Accipitridae	*Gyps bengalensis* (white-rumped vulture)	NP	NP	100 (1/1)	93 (13/14)
		*Haliastur indus* (Brahminy kite)	NP	NP	0 (0/1)	
		*Ichthyophaga ichtyaetus* (grey-headed fish eagle)	3	0	100 (4/4)	
		*Ictinaetus malayensis* (black eagle)	NP	NP	100 (1/1)	
		*Milvus migrans* (black kite)	1	0	NP	
		*Spilornis cheela* (crested serpent eagle), n = 1	1	0	100 (5/5)	
		*Spizaetus cirrhatus* (changeable hawk eagle), n = 1	0	0	100 (2/2)	
Galliformes	Numididae	*Agelastes sp* (guineafowl)	NP	NP	33.3 (1/3)	36 (5/14)
	Phasianidae	*Pavo muticus* (green peafowl)	0	3	100 (3/3)	
		*Gallus gallus* (red jungle fowl)	NP	NP	12.5 (1/8)	
Gruiformes	Gruidae	*Grus antigone* (Sarus crane)	3	0	NP	
Passeriformes	Corvidae	*Corvus macrorynchos* (large-billed crow), n = 1	2	0	100 (3/3)	25 (3/12)
	Sturnidae	*Gracula religiosa* (hill mynah)	NP	NP	0 (0/3)	
		*Acridotheres tristis* (common mynah)	NP	NP	0 (0/4)	
		*Acridotheres javanicus* (white-vented mynah)	NP	NP	0 (0/1)	
		*Sturnus contra* (Asian pied starling)	NP	NP	0 (0/1)	
Pelecaniformes	Anhigindae	*Anhinga melanogaster* (oriental darter)	0	1	NP	
	Pelecanidae	*Pelecanus philippensis* (spot-billed pelican)	3	2	NP	
Psittaciformes	Psittacidae	*Psittacula eupatria* (Alexandrine parakeet), n = 1†	1	0	50 (1/2)	0 (1/146)
		*Psittacula roseate* (blossom-headed parakeet)	NP	NP	0 (0/20)	
		*Psittacula alexandri* (red-breasted parakeet)	NP	NP	0 (0/20)	
		*Psittacula finschii* (grey-headed parakeet)	NP	NP	0 (0/104)	
Strigiformes	Strigidae	*Bubo nipalensis* (spot-bellied eagle owl), n = 1	0	0	100 (1/1)	92 (12/13)
		*Ketupa ketupu* (buffy fish owl)	NP	NP	100 (3/3)	
		*Ketupa zeylonensis* (brown fish owl)	NP	NP	86 (6/7)	
		*Strix seloputo* (spotted wood owl)	NP	NP	100 (2/2)	
	Tytonidae	*Tyto alba* (barn owl)	5	0	NP	
Total		8 sampled	42	61	18.3% (44/240)	

*S1, aviary section in which cumulative mortality rate could not be estimated because exact bird population was not known and birds were difficult to observe; S2, aviary section in which captive bird population was exactly known and number of dead birds was precisely recorded; NP, species not present in S1 or S2. †Only sample that was negative for highly pathogenic avian influenza virus (H5N1); all other birds sampled were positive.

The first case, in a crested serpent eagle (*Spilornis cheela*), was reported on December 15, 2003, in S2 (Figure). On December 19, the outbreak had reached every section and continued until January 12; a total of 86 birds, representing 8 taxonomic orders and 12 families, died (Table 1). Of 7 cat species, cats from 5 species were reported sick (16/39 total cats) (Table 2). In S2, 80% of the reported bird deaths were observed from December 15 to 21. Of the 29 wild bird species kept in S2 at the beginning of the outbreak, no birds from 12 species showed signs of disease (Table 1). Mortality rates varied among the orders, 0–100% (Table 1). The only mammals present in the aviaries in S2, slow lorises (*Nycticebus* sp.), did not become ill. None of the 27 animal keepers, who were 20–50 years of age, were reported to have gotten sick.

**Table 2:** Morbidity rates for wild cats during outbreak of highly pathogenic avian influenza virus (H5N1), Phnom Tamao Wildlife Rescue Centre, Cambodia, December 15, 2003–January 13, 2004

Order	Family	Species (common name)	Cumulative morbidity rate, % (sick/at risk), no. sampled
Carnivora	*Felidae*	*Panthera leo* (lion)	100 (2/2)
		*Panthera tigris* (tiger)	80 (8/10), n= 1*
		*Catopuma temminckii* (Asiatic golden cat)	100 (2/2), n = 1*
		*Panthera pardus* (leopard)	100 (3/3), n = 1*
		*Neofelis nebulosa* (clouded leopard)	100 (1/1), n =1*
		*Prionailurus bengalensis* (leopard cat)	0 (0/16)
		*Prionailurus viverrinus* (fishing cat)	0 (0/5)
Total			41 (16/39)

*All serum samples were positive (date of collection: March 4, 2004).

Most of the birds died within a few hours without showing any clinical signs of infection. A few birds died 1–2 days after onset of clinical signs (anorexia, extreme lethargy, occasional dark green diarrhea, respiratory distress, and neurologic abnormalities). The cats were sick for 5–7 days and exhibited anorexia and lethargy but no respiratory illness.

Laboratory investigations of the organs from 8 birds sampled in December 2003 were performed (Table 1). For those birds, West Nile virus infection was ruled out by reverse transcription–PCR (RT-PCR), according to the procedure described by Lanciotti et al. (*2*). All birds sampled, except a parakeet, were positive for influenza subtype H5N1 by RT-PCR (*3*) (Table 1). Molecular characterizations of hemagglutinin (H)5 and neuraminidase (N)1 were performed from the influenza virus (H5N1) strains from PTWRC as previously described (*4*). H5 amino acid sequences were identical in the coding region to the sequence of isolates obtained from poultry cases in Cambodia (ill poultry from a flock with high mortality rates) and similar (>96.5%) to HPAIV (H5N1) strain H5 sequences from Vietnam and Thailand in 2004 (data not shown). All belonged to the H5 clade 1 (*4*). Amino acid sequences from N1 from Cambodia were very close to each other (>97.12% identity) and to 2004 Vietnamese and Thai N1 sequences (>96%) (data not shown). The HA and NA sequences of the isolates were deposited in GenBank (accession nos. ISDN186319–ISN186324, ISDN186329, ISDN186330–ISDN186665, and ISDN242365).

Retrospective investigation of the villages surrounding the PTWRC and Phnom Penh showed that chickens from 2 flocks in which deaths had been reported in mid-December had been provided to the PTWRC, either for the restaurants or for the captive animal feeding. Furthermore, at the time of the outbreak, many wild crows were found dead in the forest surrounding the PTWRC.

The 4 cat serum samples, each from a different species, were positive for HPAIV (H5N1) with serum neutralization test (*5*); titers ranged from 10 to 40 (Table 2). None of the affected cats died.

## Conclusions

The sources of introduction of HPAIV (H5N1) within the PTWRC were probably multiple: virus-infected chicken bought to feed the carnivorous species, infected live chickens brought to restaurants near S2 (i.e., the first place where deaths were detected), and contact between infected wild and captive birds. The introduction through infected chickens is supported by the absence of an outbreak at the PTWRC after the feeding of chickens to carnivorous species was discontinued; however, deaths in domestic poultry continued in the area. In addition, almost all carnivorous bird species in S2 died (93% of Falconiformes and 92% of Strigiformes) as did most species usually fed chicken meat in captivity (herons, storks, crows, great hornbill, pelican). Diet was also the origin of the outbreak among tigers and leopards in Thailand (*6*,*7*). The dispersion of the disease between PTWRD sections was probably due to poor biosecurity measures.

The clinical outcome of wild birds with suspected HPAIV (H5N1) infection at PTWRC ranged from severe illness and death to complete absence of clinical signs, as described (*8*). Several species from the orders Ciconiiformes, Galliformes, Passeriformes, Gruiformes, Coraciiformes, and Pelecaniformes were affected during the outbreak. This observation is consistent with data published earlier, except for Coraciiformes represented by 1 bird in our study (*9*). Only the carnivorous species (*Corvus macrorynchos*) among the 5 species of Passeriformes in the aviaries showed clinical signs and later was confirmed by RT-PCR to be positive for HPAIV (H5N1). This outbreak confirms that Falconiformes and Strigiformes are sensitive to HPAIV (H5N1) infection and disease (*10**–**12***)** and shows that numerous species of these orders can be affected by HPAIV (H5N1) (Table 1). Psittaciformes and Columbiformes were not visibly affected by the outbreak although they were kept in large numbers in S2, where large numbers of deaths occurred. As non–water-bird species, they do not belong to groups in which avian influenza is commonly reported (*13*). Anseriformes, represented in PTWRC by only 4 birds (*Anas poecilorhyncha*), did not show any clinical signs. Heterogeneity in the susceptibility of wild ducks to HPAIV (H5N1), including asymptomatic infection, has been demonstrated (*14*); this species also belongs to the group of wild ducks found asymptomatically infected with HPAIV (H5N1) in the People’s Republic of China during the winter of 2005 (*15*).

The serologic evidence of influenza virus (H5N1) infection in 4 species of wild cats is in agreement with previous infection in Thailand (*6*,*7*). The report of illness in the Asiatic golden cat (*Catopuma temminckii*) and the clouded leopard (*Neofelis nebulosa*) broadens the host range of the virus among mammals.

This report confirms the great variability of wild bird and mammal responses to HPAIV (H5N1) infection. It also confirms the broadening range of susceptible species that may be specific to this clade 1 virus.
